# Vertical optical complexity shaped by submerged macrophytes

**DOI:** 10.1038/s41598-024-55824-w

**Published:** 2024-03-01

**Authors:** Viktor R. Tóth

**Affiliations:** National Laboratory for Water Science and Water Security, Balaton Limnological Research Institute, Tihany, 8237 Hungary

**Keywords:** *Potamogeton perfoliatus*, Plant architecture, Irradiance, Morphology, Photophysiology, Lake Balaton, Ecological networks, Ecophysiology, Freshwater ecology, Limnology, Environmental impact

## Abstract

The influence of macrophytes on the optical environment of the littoral zone was assessed by studying the effect of monospecific *Potamogeton perfoliatus* on the quantitative and qualitative properties of light and the response of plants to this altered environment. *P. perfoliatus* was shown to alter the optical environment and consequently its own architecture: in high-density pondweed patches, 67 percent of incident light was absorbed in the top 10 cm, while spectral properties of light was significantly altered. Leaf morphology and photophysiology adapted to these changes, with photosynthetically active biomass concentrated in the upper water layer and stem biomass increasing in the basal parts due to self-shading. This study highlights the importance of submerged macrophytes in shaping the optical environment and ecological dynamics of littoral zones. Not only do pondweed plants from different sites show very similar vertical patterns of morphological and physiological parameters, but they also contribute to similar vertical spatial variability in water optics, thus increasing habitat complexity. This added optical heterogeneity not only increases the diversity of the littoral zone, but also enriches the entire aquatic ecosystem of shallow lakes by providing additional optical ecological niches.

## Introduction

The interactions between an organism and its environment are complex, particularly in the plant kingdom. Plants, due to their sessile nature, large size, and specific phenology, can have a significant impact on their surroundings, which are constantly shifting in space and time. All organisms, including plants and submerged macrophytes, require a suitable environment for optimal growth and development. However, their growth induces modifications in the surrounding environment and simultaneously with the change of environmental conditions, as a feedback loop, the plants undergo alterations concomitant with this changing environment.

As proficient eco-engineers, macrophytes contribute significantly to the alteration of their environment for their own benefit^1,2^. Nonetheless, extensive (density-driven) macrophyte growth may not always benefit the plants themselves. An example of the complex interaction between plants and their environment can be seen in the way macrophytes modify the optical environment. As macrophytes grow, the optical environment undergoes noticeable changes, highlighting the multifaceted relationship between plants and their dynamic ecological surroundings.

Solar radiation is a fundamental component of the biosphere and is particularly important in aquatic ecosystems^3,4^. The littoral zone, which is directly linked to solar radiation, represents the near-shore habitat where light penetrates the lake bottom in sufficient quantities to support photosynthesis, therefore serving as home of macrovegetation in lakes. The euphotic zone, where aquatic plants can thrive, is a layer of water with light higher than 1–2% of the incident surface irradiance^5,6^, a principle that also applies to Lake Balaton and its macrophyte species^7^. However, less is known about how macrophytes affect spectral properties of light and how light quality affects these plants^8,9^.

Solar radiation in aquatic environments is attenuated by several physical (water depth, suspended matter, etc.), chemical (dissolved organic matter) and biological (aquatic autotrophs) factors^4,10^. Macrophytes can reduce the amount of light entering the water column by absorbing and scattering light, creating a shaded area around themselves and their neighbours, although there is wide variation in the degree of light attenuation between species^8,11,12^. Trait ecology of freshwater submerged rooted macrophytes seeks to explain the relationships between plants and their environment using functional traits^13,14^, and its approach allows comparison between species and communities^15,16^. Rooted submerged macrophytes, such as perfoliate pondweed (*Potamogeton perfoliatus* L.), due to their vertical position in the water column have a unique exposure to solar radiation, as an individual plant can experience both excess of solar radiation at its apical part and a deficiency at its basal part^8^. To maximise their ability to assimilate sunlight and convert it into chemical energy under all light conditions, submerged macrophytes have developed wide range of morphological and photophysiological traits aimed at capturing as much solar radiation as possible. Consequently, the uneven vertical distribution of radiation triggers these different morphological or photophysiological responses, ultimately leading to uneven within-plant growth and redistribution of biomass. This in turn further alters the optical environment during plant growth, introducing both spatial (vertical) and temporal variability.

The cumulative effect of these morphological or photophysiological changes often results in a very different canopy architecture of *P. perfoliatus*, creating individual optical environments and niches that remain poorly understood^9^. Canopy architecture in submerged, rooted macrophytes varies between species and may also differ within a species due to specific stages of plant life or environmental conditions^17–19^. The interaction between the plants and their environment (mostly local light availability and hydrodynamics) could detrimentally affect the appearance of plants. In Lake Balaton, perfoliate pondweed is a habitat-forming macrophyte^7,20^ and the spatially and temporally varying light conditions restructure these communities, thus creating or eliminating suitable habitats for other aquatic organisms^21,22^. This habitat complexity is a fundamental driver of littoral biodiversity. The spatial variability of aquatic angiosperms, including intra- and interspecific morphological differences between neighbouring macrophytes, contributes significantly to habitat heterogeneity^23,24^. Macrophytes, with their various surfaces and available spaces, serve as vital habitats for a wide range of species^21,25^. Therefore, understanding how these underwater plant-based frameworks develop and the factors that influence their formation is crucial for assessing and quantifying biological interactions and processes in the littoral zone. Throughout the growing season, macrophytes grow and dynamically alter not only their own biomass, but also that of organisms directly or indirectly connected to these aquatic plants, thereby modifying energy and carbon fluxes and, consequently, trophic pathways in the littoral zone^24,26^. To gain a deeper understanding of this architectural complexity, this study investigates the interaction between solar radiation and perfoliate pondweed by matching water column light conditions with leaf morphological and photophysiological parameters of the plants.

The objectives of this study are twofold. Firstly, to assess the optical environments of monospecific *P. perfoliatus* macrophyte stands in Lake Balaton, and secondly to characterize the vertical morphological and photophysiological variability of these plants in a moderate density macrophyte patches. To determine the within-plant morphological and photophysiological variability of perfoliate pondweed and how it changes with water depth in response to light environment, foliar morphological and photophysiological traits of each leaf along the stems of studied plants was determined and the following were hypothesised:

- Morphological foliar traits of *P. perfoliatus* correlate with depth, the spectral properties and the intensity of the light,
- Photophysiological foliar traits of *P. perfoliatus* correlate with depth, the spectral properties and the intensity of the light.

## Methods

### Lake Balaton

**Figure 1:**
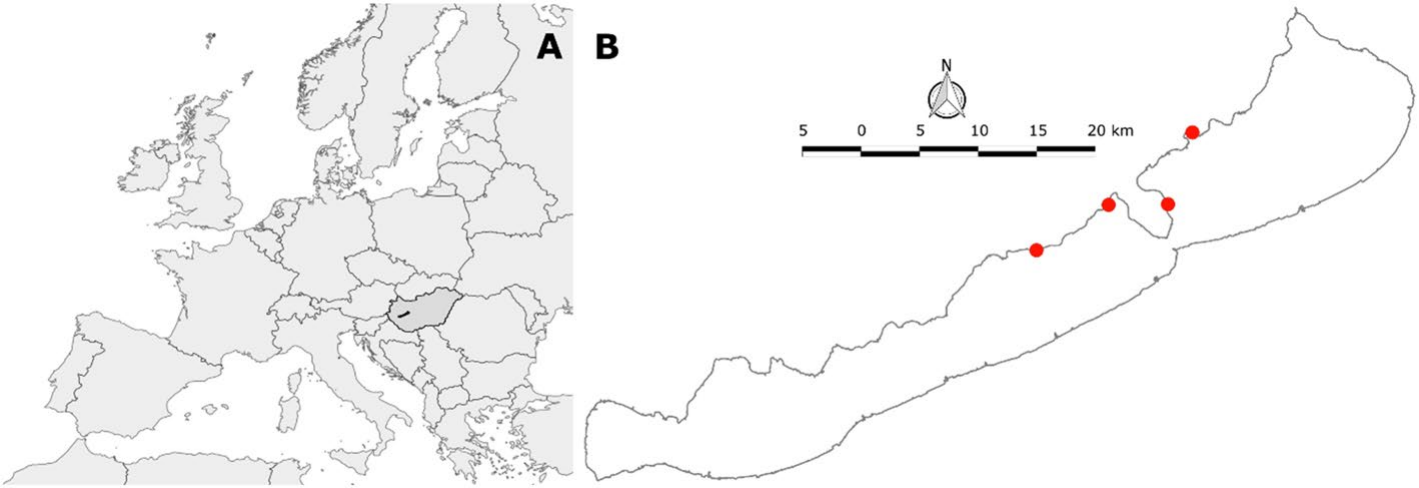
(**A**) Map of Europe showing Hungary (dark grey) and Lake Balaton (black) within it. (**B**) Lake Balaton and the sampling sites (red dots) along the northern shore of Lake Balaton. Maps were created using QGIS 3.26.2. (https://qgis.org).

Lake Balaton (Fig. 1A) is a large (596 km^2^), shallow (mean depth 3.7 m) freshwater lake located in Central Europe (N 46.83°, E 17.71°). With a length of 78 km and a width between 1.4 and 15.3 km, the lake can be divided into four basins, each of which has different limnological characteristics. The westernmost, Keszthely basin, has experienced eu-hypertrophic conditions at the end of summer due to significant nutrient inflows in the past. In contrast, the easternmost, Siófok basin, is predominantly meso-oligotrophic. The prevailing north-westerly wind direction makes the southern shore exposed to waves, while the northern shore remains relatively calm. For convenience and feasibility of the sampling, observations and measurements were made in calm weather with only moderate wavelets on Lake Balaton.

The amount of epiphyton in Lake Balaton varies considerably with time and space. To maintain consistency in the performed measurements, samplings were only performed on the northern shore of the meso-oligotrophic area, where the epiphyton had almost the same vertical distribution.

### Study areas

Observations and samplings were carried out at four sites along the northern shore of Lake Balaton (Fig. 1A,B) on multiple occasions during the peak of macrophyte phenology (end of July–beginning of August) in both 2021 and 2022. At each site, relatively large monospecific patches of *P. perfoliatus* were selected near the maximum depth of colonisation at approximately 1.7 m. These selected macrophyte stands were located in wave-exposed areas to replicate the typical conditions that prevail throughout most of the littoral zone within the meso-oligotrophic region of the lake (water chlorophyll-a 4–8 µg l^−1^, TSM—5 to 11 mg l^−1^, Secchi depth – 100–164 cm, surface water temperature 23.3 ± 0.4 °C at the time of experiments).

### Optical measurements

All optical measurements were performed from a small (< 3 m) inflatable boat. To avoid shading by the boat and the sampler, at all times the sensors were positioned at zenith and oriented to the south. All optical measurements were timed to take place around 10:30–11:30 Central European Time (CET) with solar noon at 12:54 CET.

Downwelling irradiance was measured at various points both within and outside the studied pondweed patches at all sites studied (Fig. 1B). Open water measurements were taken in close proximity (~ 2 m) to the sampled macrophyte patches, while measurements within the patches were taken at a minimum distance of 2 m from the edge of the macrophyte stand. A spherical irradiance sensor (US-SQS/L, WALZ, Germany) connected to a data logger (LI 1400, LI-COR Biosciences GmbH, Germany) was used for these measurements. Measurements were taken at 0, 10, 30, 50, 70, 90, 110, 130 and 150 cm depth with the irradiance sensor data from areas of unknown macrophyte density that latter were determined during the macrophyte sampling process. At each sampling point, 5 measurements were taken and the recorded irradiance values were averaged for each specific depth.

Spectral attenuation measurements were made both inside and outside *P. perfoliatus* patches at all sites studied (Fig. 1B). Open water measurements were taken in close proximity (~ 2 m) to the sampled macrophyte patches, while measurements inside the patches were taken at least 2 m from the edge of macrophyte stand. A spectroradiometer (USB 2000+, Ocean Optics, USA) equipped with a 10 m long, 200 μm bifurcated UV/visible optical fibre was used for measurements. For precise positioning, the optical fibre was attached to a 1 cm diameter aluminium rod, with the reading part of the fibre oriented towards the zenith and facing south. At each *P. perfoliatus* patch, 3 independent measurements were taken at specific depths (in centimetres): 0, 10, 30, 50, 70, 90, 110, 130 and 150. On each occasion and at each depth, a total of 25 spectra were automatically recorded and averaged by the proprietary software of the spectroradiometer. These independent 3 sets of spectroradiometric readings were later averaged in R software^27^.

### Biomass measurements

After optical assessment of the surveyed macrophyte patches, four 0.25 m^2^ areas of *P. perfoliatus* were harvested at each macrophyte density at the points where light attenuation was recorded. This consisted of placing a 0.5 × 0.5 m quadrant on the sediment within which all plants were carefully removed from the water by diving and cutting all plants within the quadrant at sediment level. The harvested plants were carefully washed, their quantity recorded and later bagged for transport. In the laboratory, these bagged pondweed plants were aligned by their basal parts and cut into 20 cm long segments from the basal (150–170 cm) to the near top (10–30 cm). The apical segment (0–10 cm) was sometimes longer than 10 cm because *P. perfoliatus* plants sometimes grow along the water surface. Macrophyte biomass and leaf area index (LAI_d_) were expressed in relation to the volume of the specific water layer studied, i.e. biomass and leaf area of the section were divided by 0.1 or 0.2 m^3^ (1 m^2^ area multiplied by 0.2 or 0.1 m layer height).

The harvested biomass was then separated into leaves and stems and the samples were dried at 50 °C for one week, after which the dry biomass was determined. In addition, for each depth and patch, 5 fresh leaves were scanned individually using a Canon CanoScan LiDE 60 digital scanner and then weighed after one week of drying to calculate the leaf area to weight ratio.

### Morphological and photophysiological measurements

These experiments were carried out only on *P. perfoliatus* patches with a moderate density of approximately 10 plants per square metre. At each sampling site (Fig. 1B), 5–6 intact pondweed plants were carefully selected so that the plants were undamaged and extended to the water surface, with height ranging from 1.7 to 1.8 m. Prior to measurements, periphyton was carefully removed from the leaves directly at the sampling site. One fully grown, leaf was used for each depth interval from 0–1 cm, 1–10 cm, 10–30 cm, 30–50 cm and so on. The leaves were detached from the plant just before the actual measurement.

Leaves were dark adapted for 20 min. A chlorophyll fluorimeter (PAM-2500, Heinz Walz GmbH, Germany) was used to in situ assess the chlorophyll fluorescence of the dark-adapted leaf (representing the minimum fluorescence yield—F_0_) and subsequently the maximum fluorescence yield (F_m_) of the leaf when exposed to pulse-saturated light (λ = 630 nm, Is = 3000 μmol m^−2^ s^−1^). Each leaf was then exposed to a sequence of 11 actinic illumination steps with intensities ranging from 5 to 787 μmol m^−2^ s^−1^ (duration: 15 s, λ = 630 nm). After each illumination step, the apparent (Fs) and light-adapted maximum (F_m′_) fluorescence values were measured with a new saturation pulse (λ = 630 nm, I_s_ = 3000 μmol m^−2^ s^−1^).

After fluorescence measurement, each measured leaf was in situ digitised individually using a Canon CanoScan LiDE 60 digital scanner. A 1 cm diameter disc was then excised from each leaf using a core borer. These excised leaf discs were placed on ice and transported to the laboratory in a cool box. To extract leaf pigments, leaf discs were treated with 80% acetone overnight at 4 °C. The supernatant was then subjected to absorbance measurements using a spectrophotometer (UV1600, Shimadzu, Japan). Finally, pigment concentrations were calculated using empirical equations^28^.

In the laboratory, the digital scans were analysed for various leaf morphological characteristics using ImageJ software (http://rsbweb.nih.gov/ij/). These characteristics included length, width, area, perimeter and circularity (ranging from 0 for an infinitely elongated polygon to 1 for a perfect circle).

The Study complies with local and national guidelines and regulations.

### Mathematical and statistical analysis

Plants use the ratio of red to far-red light as an environmental cue to detect the presence of nearby vegetation and adjust their growth and development accordingly. The red (650 nm) to far-red (750 nm) ratio was calculated from the recorded spectroradiometric data using the equation: r/fr = Ed_(650)_/Ed_(750)_. To estimate the spectral diffuse attenuation coefficient (Kd_(λ)_) at a given water layer Δz, the natural logarithm of the spectroradiometric measurements (Ed_(λ)_) was plotted against depth (z) and the resulting slope was used. The calculation was performed using the equation Kd_(λ,z)_ = ln[Ed_(λ,z)_/Ed_(λ,z+Δz)_]/Δz.

Chlorophyll fluorescence measurements provide valuable information on the physiological status of plants, offering insights into their photosynthetic efficiency, stress responses, and overall health. The electron transport rate (ETR) and other key chlorophyll fluorescence parameters (Table 1) were calculated from the chlorophyll fluorescence data^29–31^. The ETR response to light was fitted with an exponential saturation curve^32^, providing values for the maximum electron transport capacity (ETR_max_), the theoretical saturation light intensity (I_k_), and the maximum quantum yield for whole-chain electron transport (α). Other photochemical parameters were also derived from the data obtained (Table 1).

**Table 1:** Fluorescence parameters derived from PAM fluorometry, including equations for minimum (F_0_) and maximum (F_m_) fluorescence yields, apparent (F_s_) and maximum (F_m′_) fluorescence values, irradiance (I) and empirical absorption factor (AF = 0.84). Further details can be found in the cited literature.

Parameter	Name	Equation	References
*F*_*v*_*/F*_*m*_	Maximum quantum efficiency of PSII	(*F*_*m*_ − *F*_*0*_)/*F*_*m*_	^31^
*qP*	Photochemical quenching	(*F*_*m*′_ − *F*_*s*_)/(*F*_*m*′_ − *F*_*0′*_)	^19^
*qN*	Non-photochemical quenching	*1 − *(*F*_*m*′_ − *F*_*0*′_)/(*F*_*m*_ − *F*_*0*_)	^19^
*ETR*	Electron transport rate	(*F*_*m*′_ − *F*_*s*_)/(*F*_*m′*_)*IAF*·*0.5*	^30^

In this study, the potential photosynthetic assimilation was derived by multiplying the ETR_max_ by leaf area measured at each macrophyte level (ETR_max_ * LA). The total potential photosynthetic assimilation capacity for each plant was calculated and, in this study, presented as a percentage contribution to the total potential photosynthetic assimilation at the plant level.

Trait variability (expressed as coefficient of variance) was calculated for each specific trait at different depths. R software version 4.0.5. was used for statistical analyses^27^. The results of two independent series of measurements (optical from one side and biotic measurements from the other) were correlated and in both cases the mean values for the specific depth were used (n − 40). Linear and non-linear correlation analyses were performed using the ggcorrplot package (https://CRAN.R-project.org/package=ggcorrplot). Of the linear and non-linear correlation tests performed, the Pearson product moment correlation did not always have the highest coefficient and the lowest significance, however it is discussed in the text.

### Ethical approval

This research did not contain any studies involving animal or human participants, nor did it take place on any private or protected areas. No specific permissions were required for corresponding locations.

### Permission to reproduce material from other sources

All data and illustrations used in the manuscript are original and have been produced specifically for this manuscript.

## Results

### Biomass distribution

**Figure 2:**
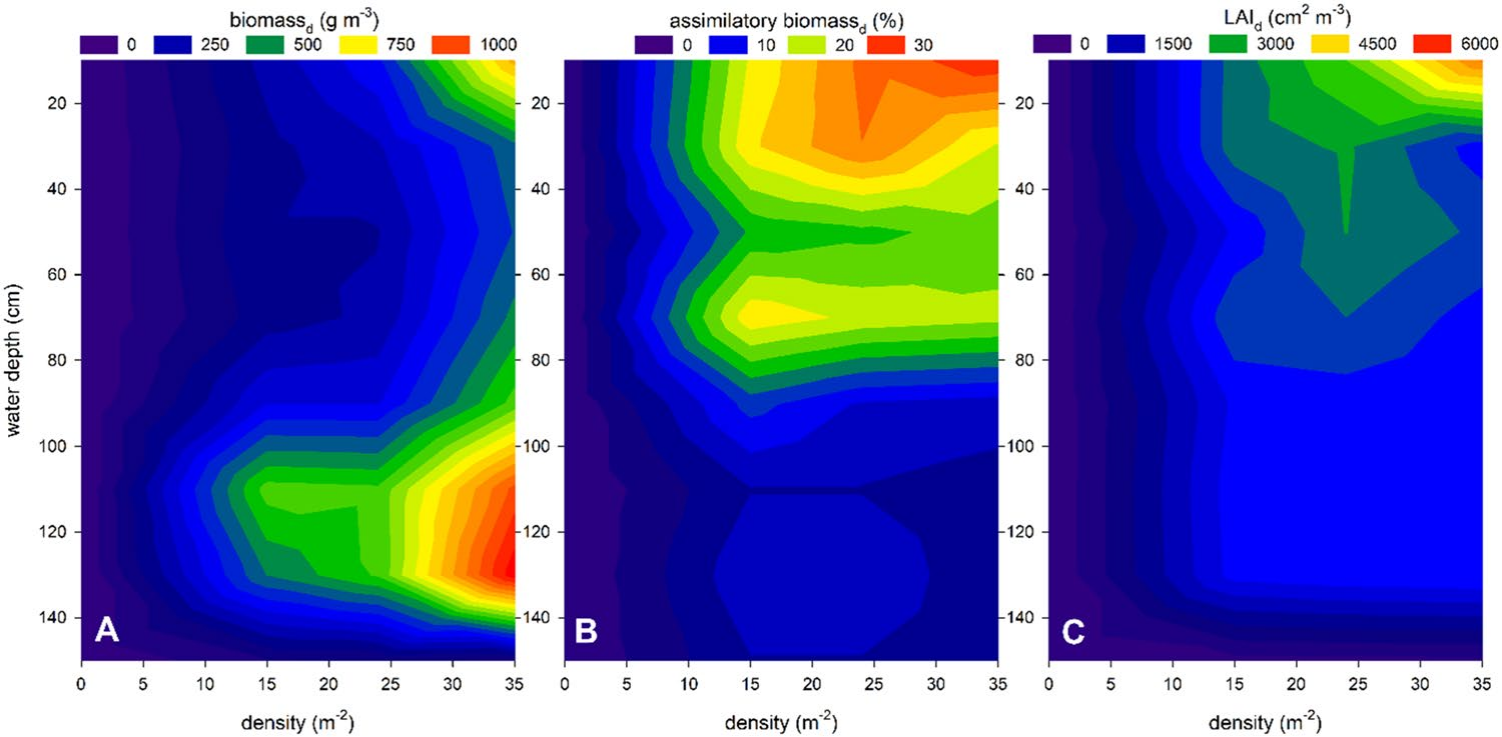
Vertical distribution of average biomass (**A**—g m^−3^), assimilatory biomass ratio (**B**—%) and leaf area index at the specific depth (**C**—cm^2^ m^−3^) in monospecific *Potamogeton perfoliatus* stands of varying density in Lake Balaton. Data are expressed in relation to the volume of the specific water layer studied.

The vertical distribution of *P. perfoliatus* plant biomass showed different patterns in macrophyte patches of different densities (Fig. 2). Low-density macrophyte patches had a relatively uniform distribution of biomass throughout the water column, with values around 270 g m^−3^. However, as macrophyte density increased, a bimodal biomass distribution was observed (Fig. 2). Specifically, biomass concentrations were evident at two depths: one near the surface with a biomass of 800 g m^−3^ per depth, and another in close proximity to the sediment with a value of approximately 1000 g m^−3^. Within these dense patches of *P. perfoliatus* near the surface, leaves represented 30% of the total biomass, resulting in significantly elevated leaf area index (LAI) values approaching almost 5000 cm^2^ m^−3^ (Fig. 2). Conversely, in the dense pondweed patches, the increased concentration of basal biomass was mainly due to stem thickening, as the leaf biomass fraction remained below 10% of the total biomass (Fig. 2).

### Optical environment

**Figure 3:**
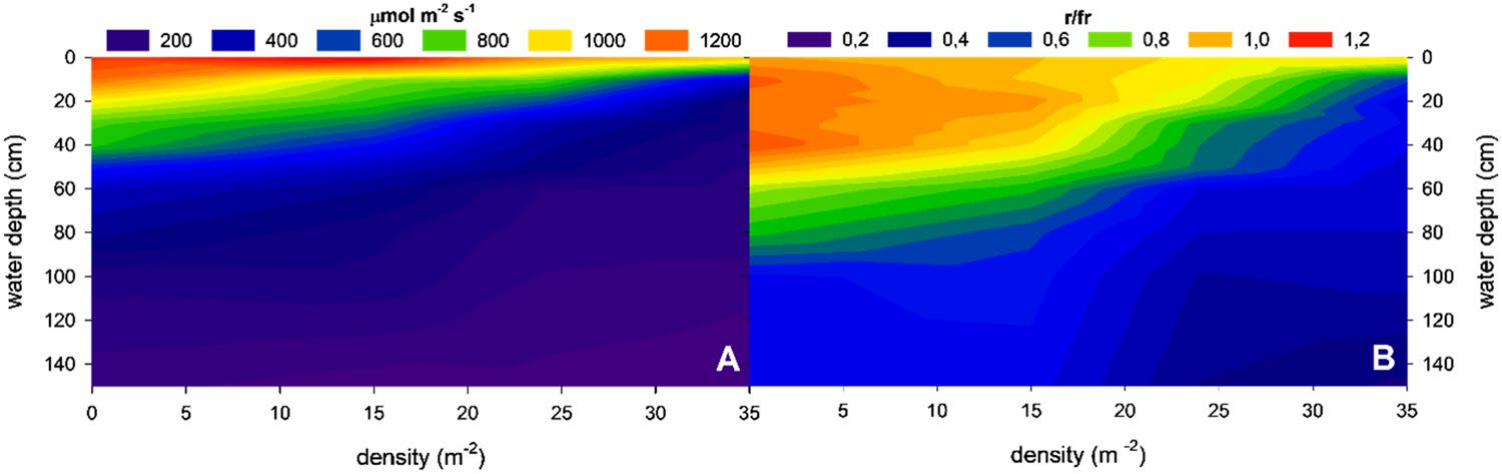
Vertical and density dependent change of average photosynthetically active radiation (**A**—I, μmol m^−2^ s^−1^) and average red to far-red light ratio (**B**—r/fr) in a monospecific *Potamogeton perfoliatus* stands in the meso-oligotrophic part of Lake Balaton.

The biomass distribution described above had a significant effect on light attenuation by macrophytes. In areas without macrophytes, the light intensity reaching the water surface within a 60 cm water column was reduced by 50% (Fig. 3). In low-density patches of *P. perfoliatus*, the depth at which light intensity was halved remained almost the same as in open water, at around 50 cm. In dense pondweed patches, a more pronounced decrease in light intensity was observed, with light reduced to a third of its original intensity within the top 10 cm (Fig. 3).

In addition to light intensity, the quality of light within the water column also changed (Fig. 3B, Supplementary Fig. 1). In open water areas, the light absorption coefficient exhibited a wavelength-dependent bimodal distribution, with light absorption peaking at 400–500 nm and 600–700 nm, respectively. This resulted in an almost unchanged red to far-red light ration (r/fr − 650 nm/750 nm) in the upper 90 cm of Lake Balaton (Fig. 3B). However, within moderately dense pondweed patches (15 plants m^−2^), there was a marked threefold increase in light absorption intensity, with the most intense light absorption (K_dλ_ − 3) observed predominantly in the 450–650 nm wavelength range. This increase in the density of macrophytes was able to reduce the layer with an unchanged r/fr ratio to 60 cm (Fig. 3B). In very dense *P. perfoliatus* patches, this light absorption increased further, occasionally reaching a value of 6 between 500 and 600 nm (Supplementary Fig. 1). The consequences of this spectral change was the dramatic shift in the ratio of red to far-red light (r/fr − 650 nm/750 nm, Fig. 3B): in dense pondweed populations, the unchanged r/fr ratio was observed much closer to the water surface, within the upper 10–20 cm (Fig. 3B).

### Morphological and physiological variability

**Figure 4:**
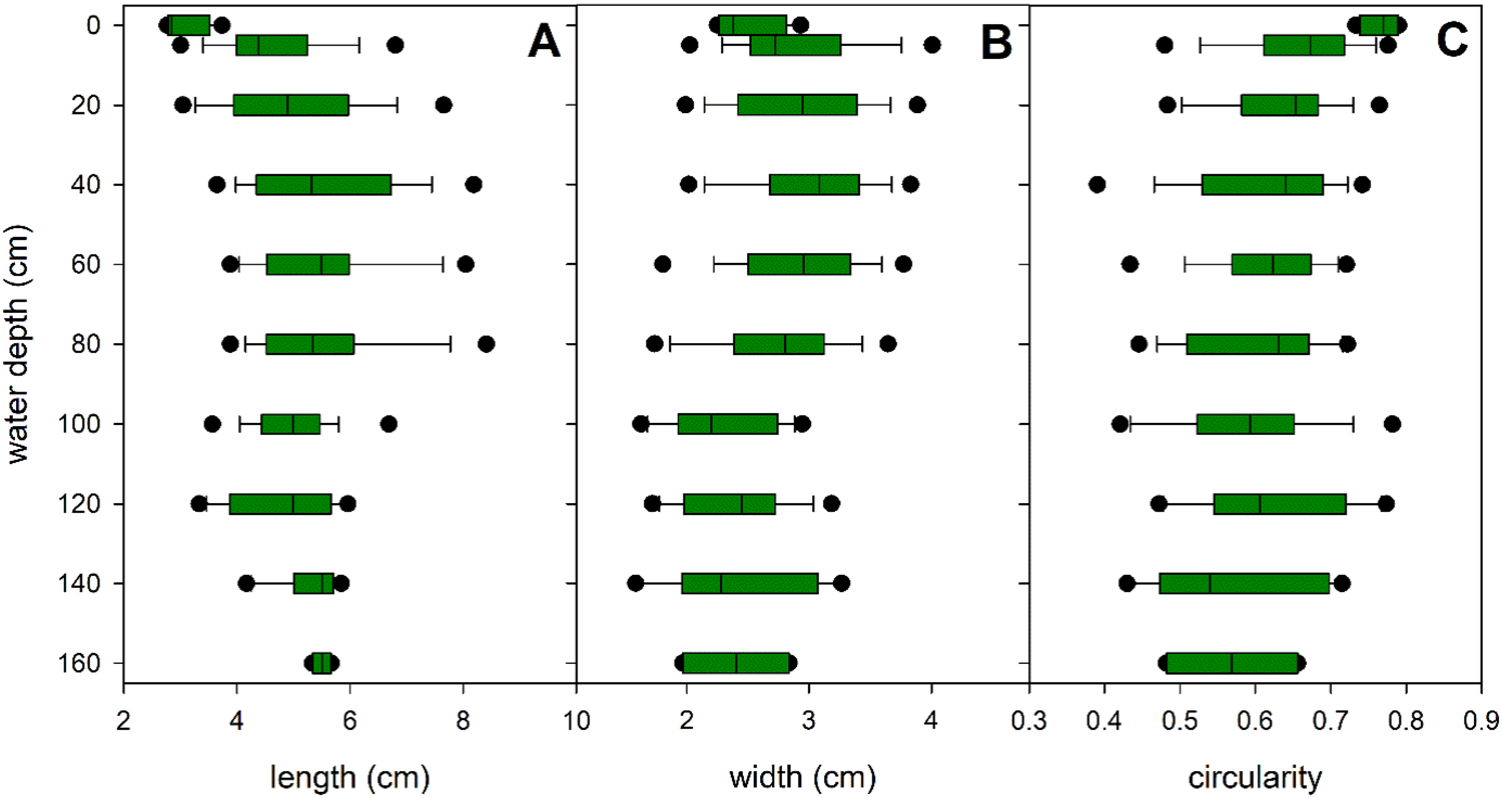
Within-plant vertical variation of leaf morphological parameters in monospecific *Potamogeton perfoliatus* stands in Lake Balaton. The figure displays the median value within the boxplot with a black line, the range containing 75% of the data with whisker caps, and the 5th/95th percentiles with black symbols.

Leaves elongated consistently between the depth of 5 cm till the basal leaves, ranging from 3.1 ± 0.5 to 5.5 ± 0.2 cm (Fig. 4A). In contrast, both apical and basal leaf width remained relatively stable at 2.4–2.5 cm, except for a slight increase in width (3.1 ± 0.6) at 40 cm depth (Fig. 4B). These parallel changes resulted in a modification of leaf shape, with apical leaves appearing almost circular, while basal leaves became significantly elongated (Fig. 4C). These morphological parameters did not show significant associations with depth, although some of them (such as leaf length and circularity) could be related to variations in light intensity and r/fr ratio within the water column (Table 2, Supplementary Fig. 2).

**Table 2:** Pearson product moment correlation (correlation coefficient and its significance—r^P^) between the studied morphological and photophysiological parameters of *Potamogeton perfoliatus* plants and their position (depth), light intensity (I) and red far-red ratio (r/fr) of the studied leaves within the water column in Lake Balaton.

	depth		r/fr		I	
	r	*P*	r	*P*	r	*P*
α	− 0.669		0.577		0.562	
ETR_max_	− 0.824	**	0.810	**	0.803	**
I_k_	− 0.743	*	0.744	*	0.732	*
F_v_/F_m_	0.766	*	− 0.879	**	− 0.905	**
qN	− 0.907	***	0.909	***	0.909	***
qP	− 0.905	***	0.893	**	0.883	**
length	0.559		− 0.729	*	− 0.755	*
width	− 0.348		0.287		0.292	
LA	0.217		− 0.363		− 0.375	
perimeter	0.393		− 0.585		− 0.615	
circularity	− 0.433		0.626		0.674	*
total chl	0.581		− 0.740	*	− 0.780	*
chl_a_/chl_b_	0.558		− 0.722	*	− 0.763	*
chl/car	0.596		− 0.751	*	− 0.789	*

The shown parameters are the maximum quantum yield for whole-chain electron transport (α), maximum electron transport capacity (ETR_max_), the theoretical saturation light intensity (I_k_), maximum quantum efficiency of PSII (F_v_/F_m_), non-photochemical quenching (qN), photochemical quenching (qP), length, width, leaf area (LA), perimeter and circularity of leaves, total chlorophyll (total chl), chlorophyll a to b ration (chl_a_/chl_b_), total chlorophyll to carotenoid ratio (chl/car). The significance of the correlations: *P < 0.05, **P < 0.01, ***P < 0.001.

**Figure 5:**
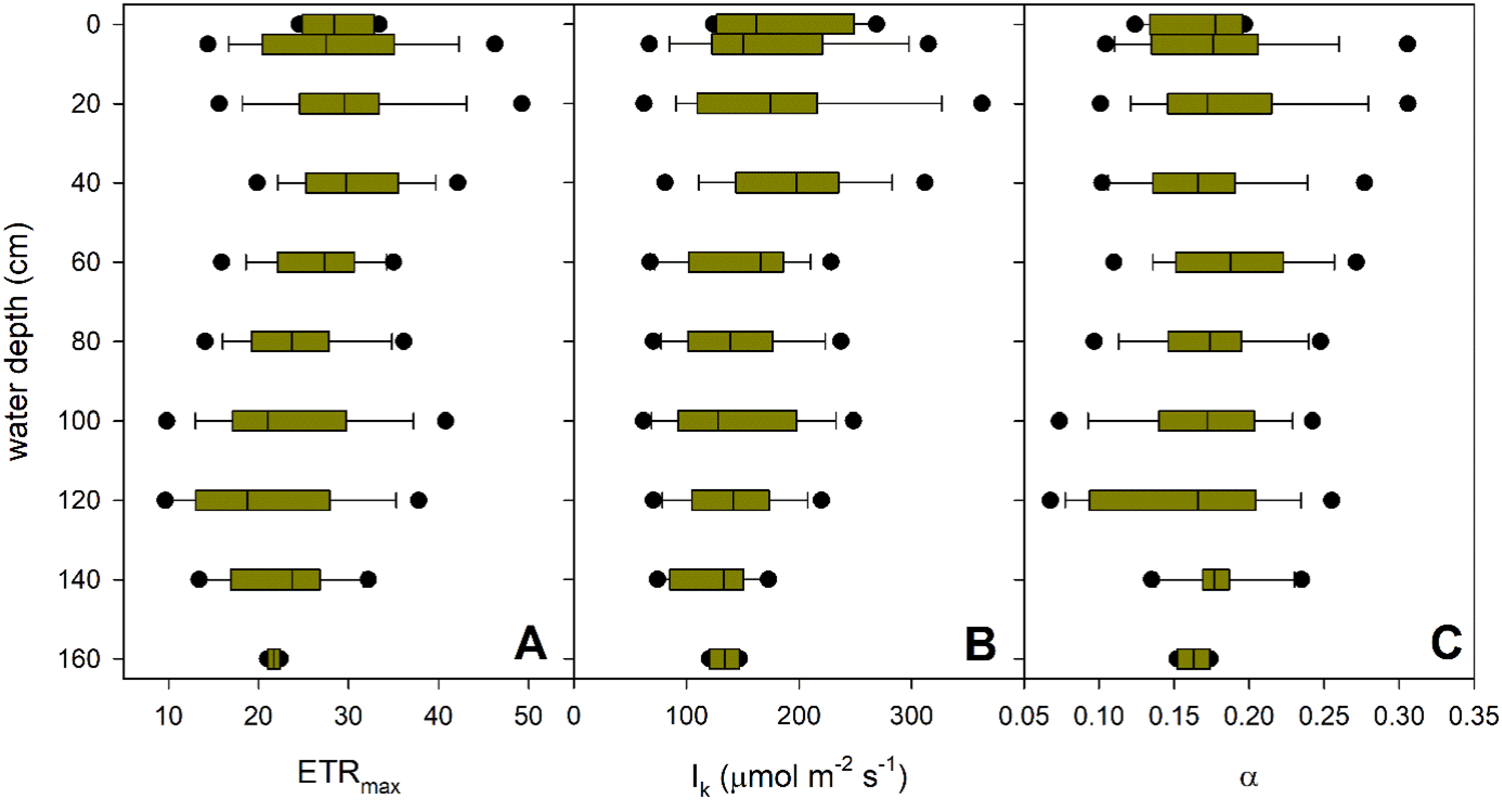
Within-plant vertical variation of foliar photophysiological parameters in monospecific *Potamogeton perfoliatus* stands in Lake Balaton. The figure displays the median value within the boxplot with a black line, the range containing 75% of the data with whisker caps, and the 5th/95th percentiles with black symbols.

**Figure 6:**
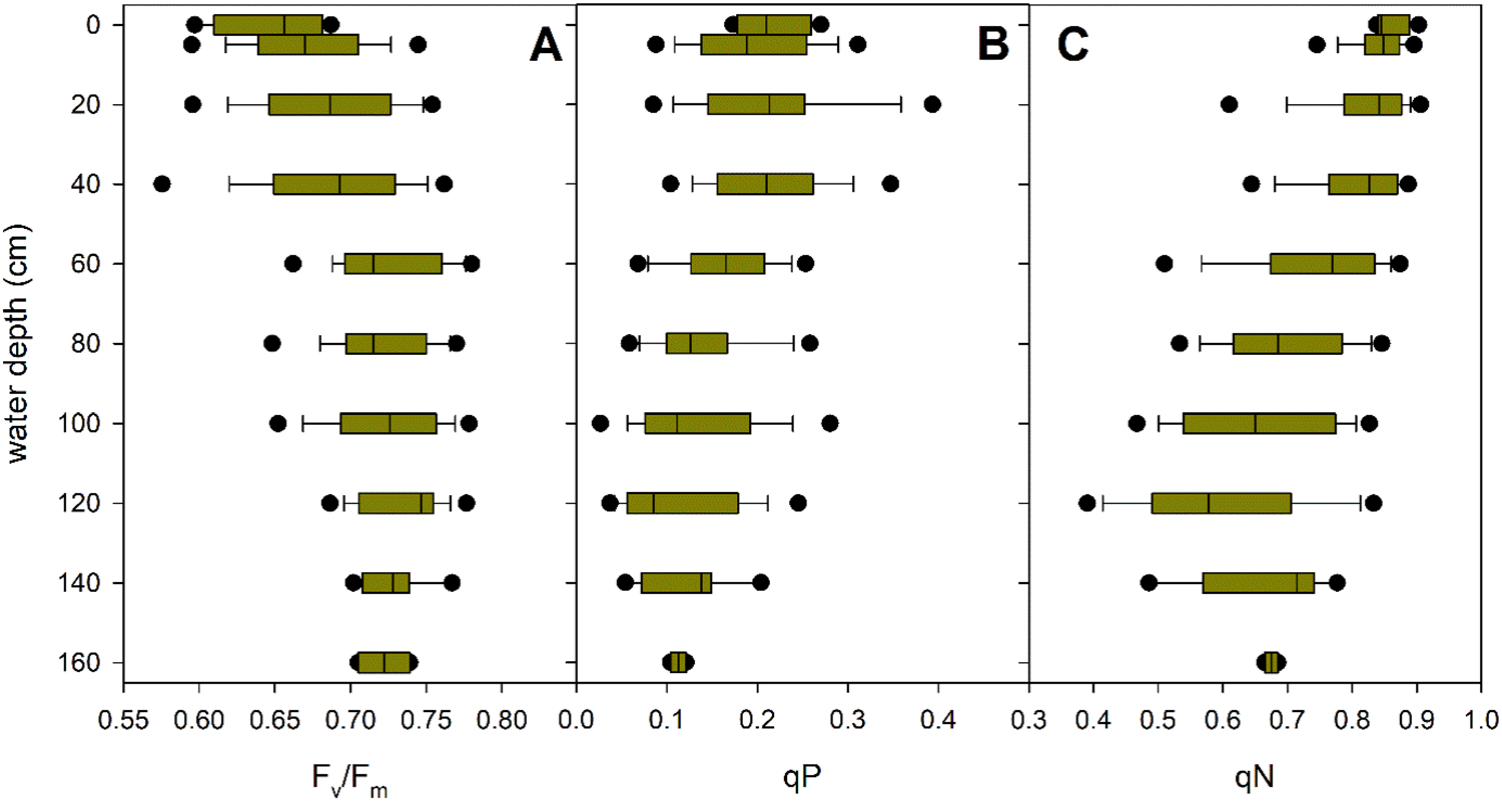
Within-plant vertical variation of foliar photophysiological parameters in monospecific *Potamogeton perfoliatus* stands in Lake Balaton. The figure displays the median value within the boxplot with a black line, the range containing 75% of the data with whisker caps, and the 5th/95th percentiles with black symbols.

In contrast to the morphological parameters of *P. perfoliatus* plants, the photophysiological parameters showed a remarkable vertical variation (Figs. 5, 6, Table 2). With increasing depth, the light absorbed and electron transport decreased, resulting in a decrease in the initial slope of the light-limited part of the light response curve (α), photosynthetic rate (ETR_max_) and saturating light intensity (I_k_) (Fig. 5, Table 2). On average, there was a 27% decrease in ETR_max_ and 30% decrease in I_k_. In addition, the decrease in light availability with depth, coupled with the lower r/fr ratio, also affected other photochemical parameters, indicating a decrease in oxidative stress within the leaves in the basal part of the studied plants (Fig. 6, Table 2, Supplementary Figs. 3 and 4).

**Figure 7:**
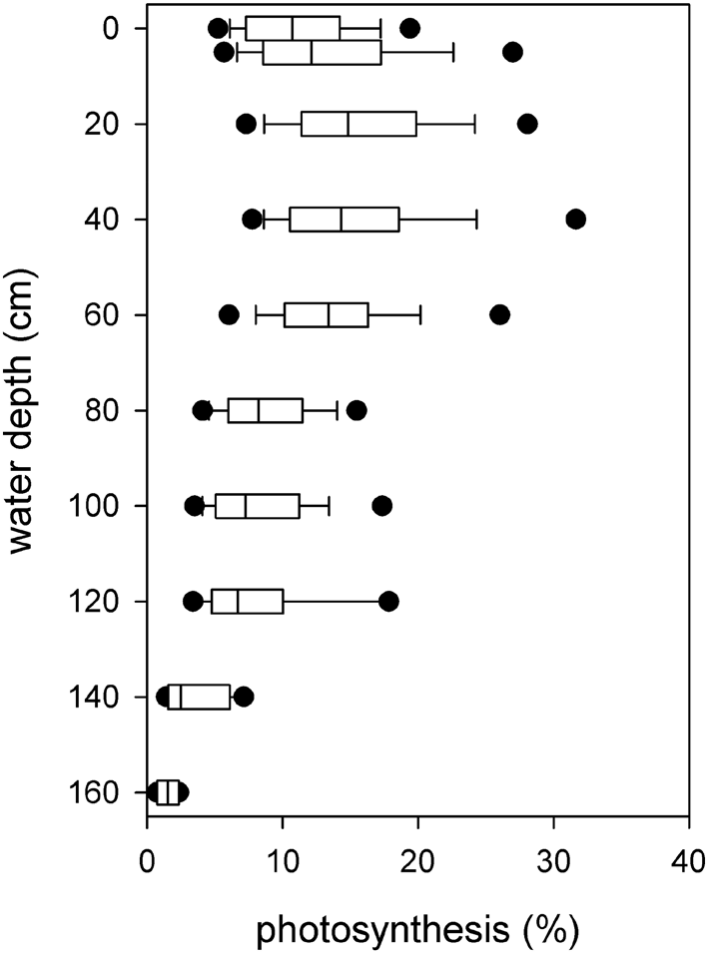
Within-plant vertical distribution of total potential photosynthetic assimilation in monospecific *Potamogeton perfoliatus* stands in Lake Balaton. The figure displays the median value within the boxplot with a black line, the range containing 75% of the data with whisker caps, and the 5th/95th percentiles with black symbols.

Based on the morphological and photophysiological parameters obtained, the theoretical assimilation capacity of an average pondweed plant was determined by calculating the product of the theoretical maximum photosynthetic capacity and the leaf area (ETR_max_ * LA). The distribution of photosynthesis within the *P. perfoliatus* plant showed a distinct vertical arrangement (Fig. 7). It is noteworthy that this uneven distribution was observed despite the fact that sufficient light (greater than the theoretical whole plant saturation light intensity I_k_ = 160 ± 67) was available for photosynthesis throughout the water column (Fig. 3A). The parts of the plant in the upper 40 cm of the water column showed a higher photosynthetic potential compared to the remaining 120 cm of the plant (56% vs. 44%, Fig. 7).

**Figure 8:**
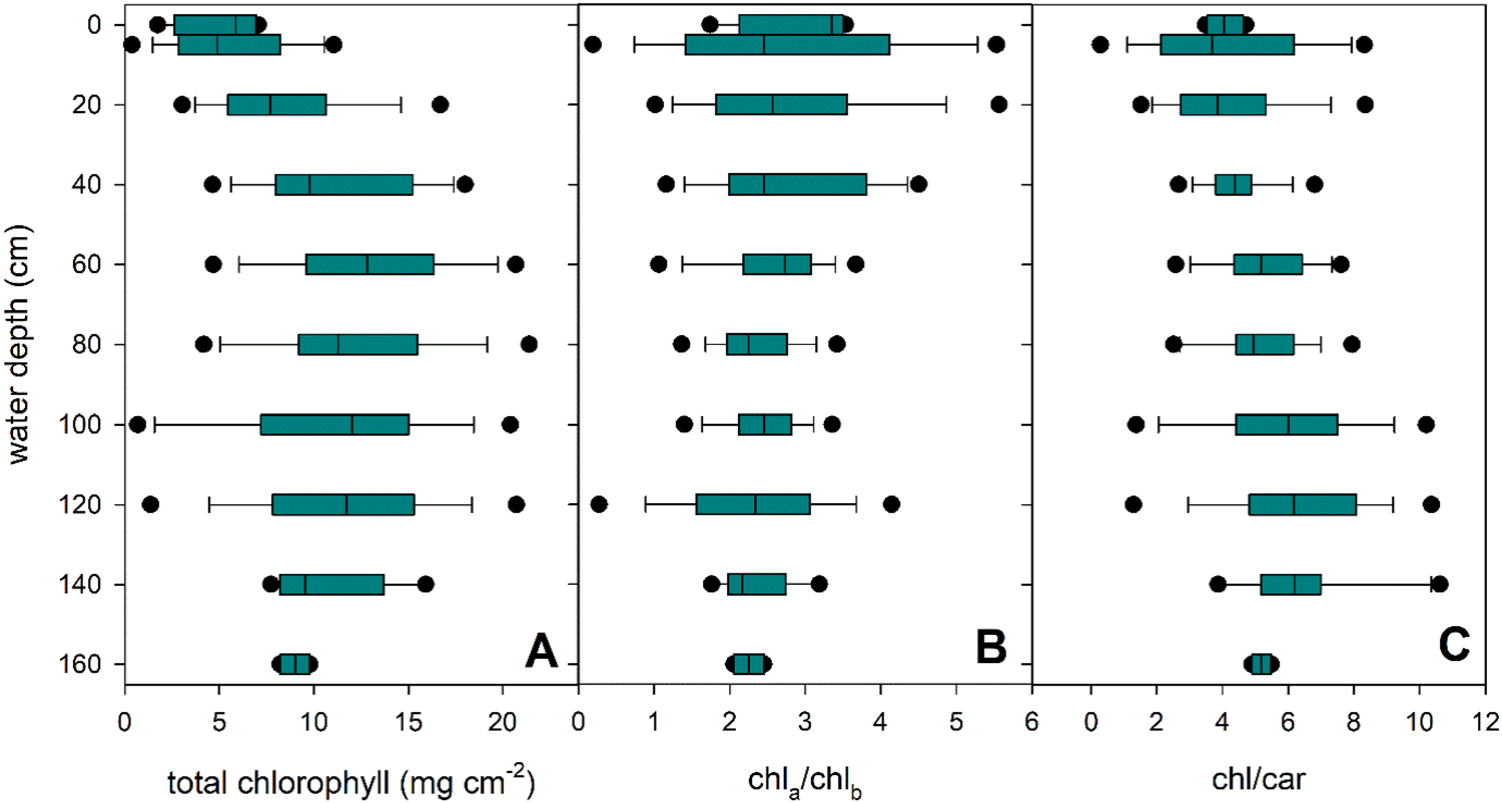
Within-plant vertical distribution of leaf pigment content in monospecific *Potamogeton perfoliatus* stands in Lake Balaton. The figure displays the median value within the boxplot with a black line, the range containing 75% of the data with whisker caps, and the 5th/95th percentiles with black symbols.

Pigment content of *P. perfoliatus* leaves showed vertical variation within the plant (Fig. 8, Table 2). The most significant changes in total chlorophyll content occurred in the apical part of the plants within the upper 60 cm of the water column, where the chlorophyll content of the leaves increased by a factor of 2.5 (Fig. 8A) and remained almost the same until the basal part of the plant. In contrast, pigment ratios showed a gradual change from the apical to the basal part of the plant, with a 25% decrease in the chlorophyll a/b ratio and a 56% increase in the chlorophyll/carotenoid ratio (Fig. 8). These changes correlated mainly with light intensity and quality (red/far-red ratio of light) (Table 2, Supplementary Fig. 5).

**Figure 9:**
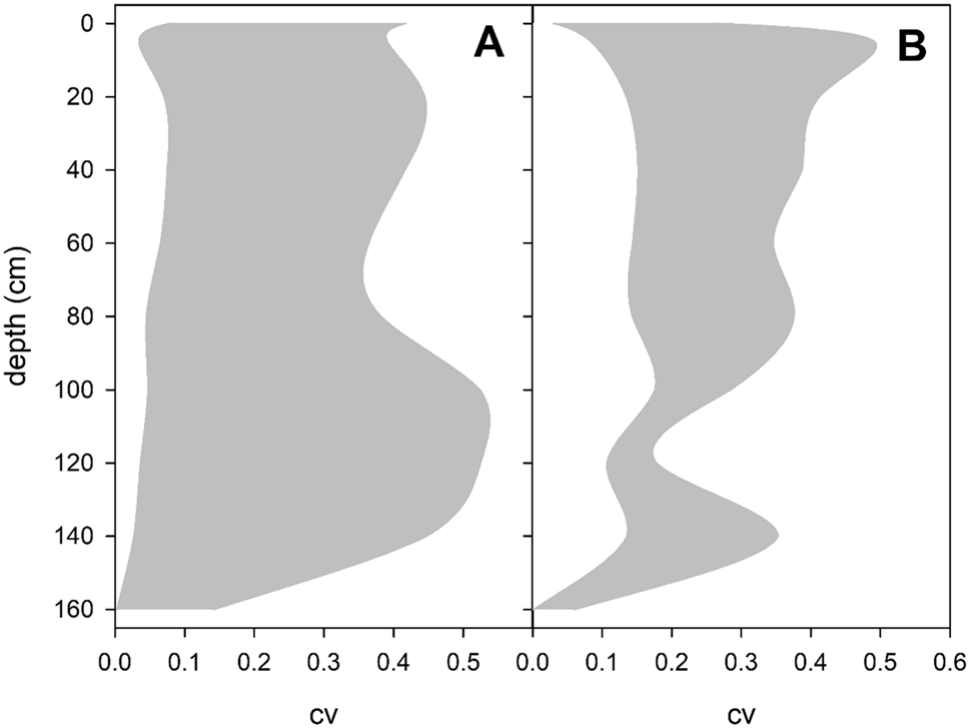
Vertical distribution of trait ranges calculated as coefficient of variability of photophysiological (**A**) and morphological and pigment (**B**) foliar parameters in monospecific *Potamogeton perfoliatus* stands in Lake Balaton.

Most of the traits, except F_v_/F_m_, showed a moderate to high variability of about cv ~ 0.25. The variability of photophysiological traits showed a bimodal vertical distribution, with smaller peaks (average cv ~ 0.27) observed at 20 cm below the water surface and larger peaks (average cv ~ 0.36) found in the basal parts of the pondweed plants at 100–140 cm depth (Fig. 9A). In contrast, the variability of morphological traits and leaf pigment peaked (cv ~ 0.28) at 20–40 cm below the water surface and gradually decreased towards the basal part of the plants (Fig. 9B).

**Figure 10:**
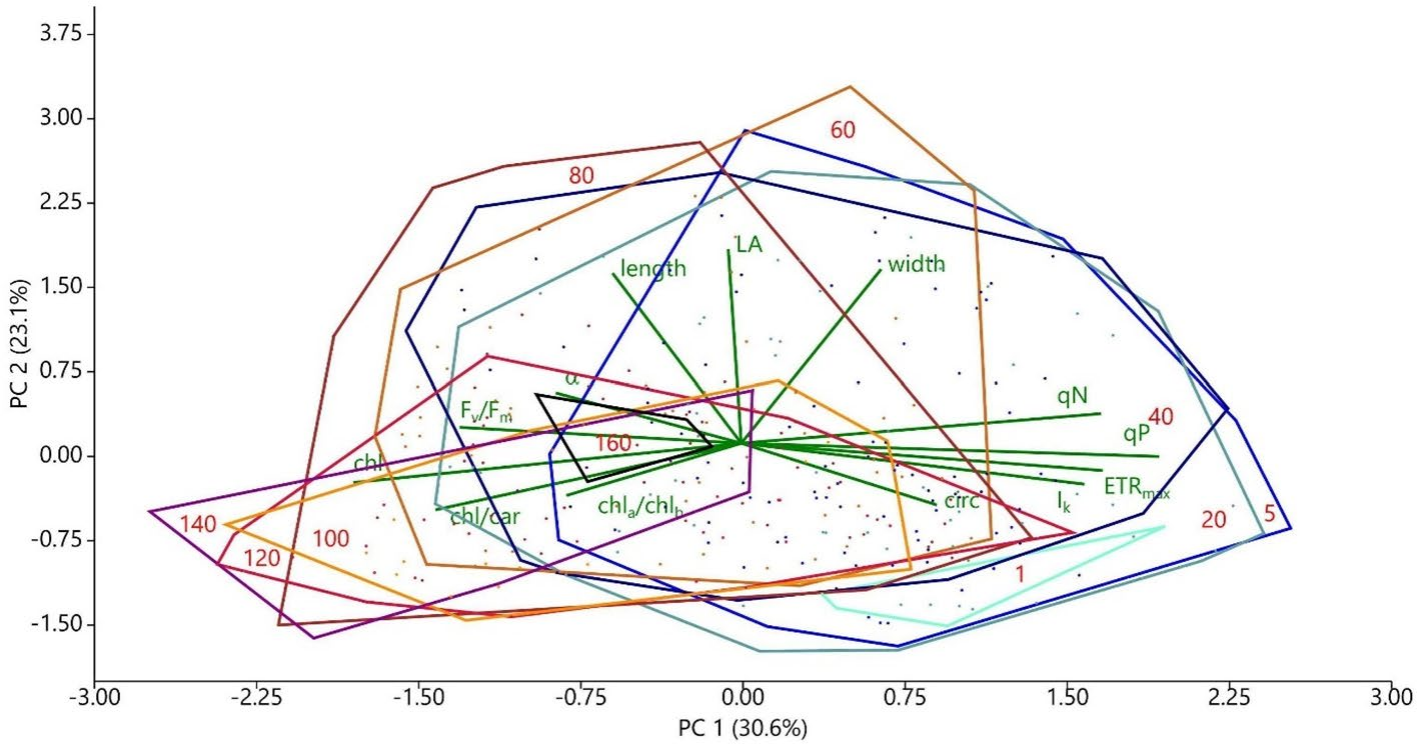
Principal component analysis of all studied foliar parameters in monospecific *Potamogeton perfoliatus* stands in Lake Balaton.

Principal component analysis of all investigated leaf traits of *P. perfoliatus* showed the gradual shift of parameters with depth (Fig. 10). The observed changes along axis 1, which also accounted for 30.6% of the total variability of the data, were mostly photophysiological, leaf pigment traits, thus placing samples from 1, 5, 20 and 40 cm deep water layers on one side of the spectrum and leaf traits from 100, 120 and 140 cm depth on the other. Changes in morphological traits were primarily associated with axis 2, which accounted for 23.1% of the variability in the data (see Fig. 10). Morphological parameters at depths of 60–80 cm and 20,140 cm were located at opposite ends of the range, however, significant overlaps were present in morphological data.

## Discussion

**Figure 11:**
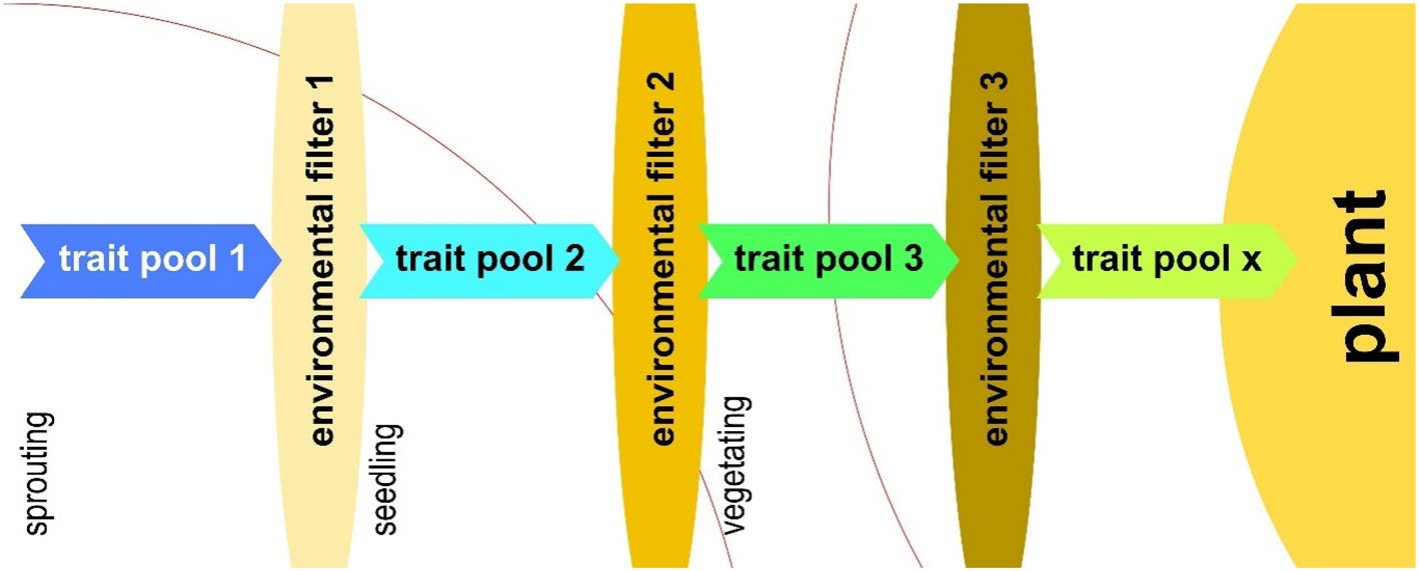
Conceptual framework for the formation of plant appearance resulting from the interaction between macrophyte traits and the environment.

The relationship between macrophytes and the environment in aquatic ecosystems is a dynamic and interconnected process. Changes in one the aspects influences the other in multiple feedback loops. In the water column of a freshwater rooted submerged macrophyte patch, several components attenuate incident light. On the one hand, some of these attenuation mechanisms could be considered passive or stochastic, such as total suspended matter and coloured dissolved organic matter in the water. These mechanisms cannot be predicted precisely due to their nature. However, predictable attenuation mechanisms could be classified as active since their attenuation is affected by the environmental conditions and due to the significant overlap in response-effect traits of the epiphytic algae and macrophytes themselves these mechanism could actively modify environment (Fig. 11). Furthermore, the transition sequence from 'environmental filter 1' to 'environmental filter 3' follows a pattern determined by the phenological shift of periphytic algae and submerged macrophytes (Fig. 11).

Light is a crucial factor for macrophyte growth, as it serves as the primary energy source for photosynthesis. In the first part of this work density of the *Potamogeton perfoliatus* plants was the studied influencing factor affecting optical environment in the surroundings of submerged macrophytes. It was found that in low-density stands, as individual plants were more spaced out, allowing more light to penetrate through the water column, the light attenuation was low, more reminiscent of the open water light attenuation as the fewer leaves and stems did not obstructed the passage of light. As the density of patches increased so does the light attenuation as the more densely packed plants increased competition for light among the individual plants and within the plants, affecting all the communities nearby^33–35^. Besides the well-documented light attenuation, also a pronounced spectral change of light was registered. This study and previous works^8^ show that photosynthetic pigments of macrophytes and periphyton absorb specific wavelengths of the incident light, thereby reducing its penetration through the water column. These spectral changes could alter the morphology, development and growth patterns of the plants themselves^9,36^, and influence other organisms in different way^37,38^. Absorption of photosynthetically important red light by macrophytes resulted a change in the ratio of red to far-red light within the water column: in low density patches the ratio was nearly as in open water, while in high density patches the ratio decreased by 50% within centimetres. Previous studies found that epiphytic algae colonising macrophyte surfaces exert a significant influence on light attenuation^8^, while the macrophytes too affect the epiphyte communities^39^.

The macrophyte germinating in an optimal environment will actively modify this environment during its ontogeny (Fig. 11), and in response the modified environmental conditions (environmental filter 2, 3 or x) will influence the growing, developing organism. This interplay will influence the appearance of the plant and manifest itself in its specific architecture.

The vertical, within-plant distribution of *P. perfoliatus* biomass was found to be density-dependent, resulting in distinct biomass distribution patterns and significant variation in biomass and leaf area across the water column. In patches with low plant density, total biomass and foliar biomass were evenly distributed throughout the water column. However, as plant density increased, especially in higher density patches, a quantitative and qualitative redistribution of biomass occurred. Specifically, photosynthetically active biomass became focused in the upper layers (top 40 cm) of the water column, while structural plant biomass (stem) increased in the basal part of the plants. The shift in biomass distribution had a dual nature. On the one hand, it represented a morphological and physiological response in the apical part of the plant aimed at maintaining or increasing the total photosynthetic capacity of the whole plant. This response was accompanied by a loss of foliar biomass due to self-shading in the basal parts of the plants. On the other hand, significant changes in the biomechanical properties of the stem were observed, leading to an increase in structural biomass to compensate for the possibly increased hydrodynamic shear stress caused by wave impact and the uneven redistribution of foliar biomass to the upper layers of the plant. These observed changes were probably triggered by the specific optical environment created by macrophyte plants at different densities.

The biomass of *P. perfoliatus* plants effectively absorbed photosynthetically active radiation, resulting in a significant reduction in light intensity over short distances. This reduction was observed as a function of macrophyte density, with high-density patches of plants halving the incident irradiance within the top 15–20 cm of the water column. In addition, the dense *P. perfoliatus* patches not only increased light absorption but also induced changes in light quality, particularly in the 450–650 nm wavelength range. Here, absorption rates were 2–2.5 times higher than in the regions below 400 nm and above 700 nm. This preferential light absorption was also evident in the red to far-red ratio (r/fr − 650 nm/750 nm). In low density plots at 50 cm depth, this ratio averaged 0.98. In contrast, in high density patches, the ratio was approximately 0.46, indicating the pronounced spectral effect of macrophytes on their environment. These changes in the optical environment subsequently led to changes in the leaf morphology and physiology of affected *P. perfoliatus* plants.

While some morphological traits such as leaf width, leaf length and leaf shape showed minor variations with water depth, they did not show any correlation with depth or light intensity and quality. Leaf area was the only trait that showed a weak correlation with light quantity and quality. This minimal response to the optical environment is not surprising given that leaf morphogenesis is a slow and relatively unresponsive process. In contrast, physiological parameters such as leaf chlorophyll fluorescence and leaf pigment content showed a remarkable responsiveness to the optical environment of the plants. Data from the study revealed significant changes in these traits with increasing depth, decreasing light intensity and decreasing red to far-red (r/fr) ratio. These responses were directly aimed at increasing the efficiency of the plant in light absorption and photosynthetic rates at all depths. Theoretical assimilation capacity, determined from photophysiological parameters and leaf area, revealed a distinct vertical imbalance in photosynthesis among pondweed plants, with the top 25% of plants contributing the majority of total photosynthesis within the plant. In addition, as a notable example of leaf-level sensitivity, the pigment content of *P. perfoliatus* leaves followed a distinct vertical pattern, with a significant increase in total chlorophyll content in the upper 60 cm of the water column. Pigment ratios also shifted between the apical and basal parts of the plant, reflecting an adaptation to the changing optical environment between the upper and lower layers of the water column.

Trait variability exhibited an interesting, although not very pronounced, vertical pattern showing the responsive and less responsive parts of the plant. The variability of morphological and leaf pigment traits was greater in the apical part of the plants (upper 20–40 cm of the water column) and gradually decreased (by about 50%) towards the basal parts. In contrast, the variability of photophysiological traits displayed a bimodal vertical distribution, with smaller peaks observed at 20 cm below the water surface and larger peaks found in the basal parts of *P. perfoliatus* plants. These data show two major centres of adaptation/acclimation within the plants, with the apical part of the pondweed responding at multiple levels with morphological, physiological and pigment adaptation/acclimation. These hotspots of variability allow specialisation of pondweed in different optical niches within the water column, thereby increasing the overall fitness of the individual.

There was some vertical redistribution of biomass in patches of moderate-density *P. perfoliatus* compared to the low density pondweed stands. There was a slight concentration of biomass in the basal parts of plants due to increase of stem biomass, while in the apical parts of pondweeds the more biomass was in leaves (25% instead of 10% in basal parts). The observed biomass redistribution may be linked to the vertical pattern of photophysiological traits because the upper water layers showed slightly more optimal conditions for photosynthesis. Increasing plant density resulted in a more significant redistribution of biomass with a further increase of foliar biomass (30–35%) in the upper layers of the water column. The gradual disappearance of basal leaves could be due to an optically suboptimal environment in the deeper layers of the water column. The combined effect of reduced light intensity and changes in the spectral composition of light (for example lowering of r/fr ratio) with depth, indicating that there is an insufficient amount of usable red light in the deeper parts of the water column and this decreased the photophysiological parameters of leaves making photosynthesis not sustainable.

The photosynthetic system of *P. perfoliatus* plants underwent significant restructuring with depth and at higher plant densities. While the apical leaves exhibited all the typical characteristics of sun-adapted leaves (higher I_k_, ETR_max_ and higher chlorophyll a/b ratio), with increasing depth the leaf parameters started to show more shade-adapted characteristics. This shift in photophysiological traits and pigment content with increasing *P. perfoliatus* density resulted in a redistribution of photosynthetically active leaf area towards the apical part of the plants. Despite supraoptimal light intensity (> I_k_) throughout the water column, the basal leaves of the plants became less effective and gradually disappeared. Within dense patches of *P. perfoliatus*, self-shading not only reduced the light intensity but also the photosynthetically active spectra (red light − low r/ir ratio), and these two conditions created an unfavourable optical environment for leaf photosynthesis in the deeper layers of the water column, resulting in the degradation of the basal leaves.

The patchy growth of macrophytes in the littoral zone has created complex structures and habitats that provide shelter and refuge^40,41^, serve as food sources^42,43^, and act as nurseries and breeding grounds^44,45^ for a wide range of organisms. Nevertheless, this study has shed light on another aspect of the impact of macrophytes. The presence and foliar characteristics of *P. perfoliatus* played a pivotal role in shaping the vertical pattern of the light environment within the macrophyte stands, creating an additional orthotropic dimension to the existing habitat complexity and ecological dynamics of the littoral zone. This vertical stratification could contribute to the formation of additional habitat layers, introducing spatial patchiness not only horizontally but also vertically. The availability of diverse optical microhabitats and ecological niches could support a wide range of organisms that require a specific optical environment, promoting a rich and diverse littoral ecosystem.

The littoral zone of freshwater lakes is an important habitat for many organisms and macrophytes play a crucial role in shaping it. Macrophytes modify their physical and chemical environment, increasing habitat complexity and creating a variety of niches for other organisms to occupy, providing physical structure and background for sensitive species^24,46,47^. This has a cascading effect on the whole aquatic ecosystem, affecting the distribution and abundance of other organisms outside the littoral zone.

The results of this study provide additional information on the complex interactions between the littoral environment and the macrophytes within it, highlighting the crucial role of macrophytes in shaping the optical environment of their surroundings. Previous studies have extensively documented the influence of macrophytes on habitat variability in lakes^21,23,48^; however, these studies primarily focused on horizontal attributes of spatial variability. In contrast, this study gives a unique insight on the importance of macrophytes in providing the vertical aspect of habitat complexity in the littoral zone.

### Supplementary Information

Supplementary Figures.

## Data Availability

Macrophyte morphological and photophysiological data will be available for non-commercial purposes after acceptance of the manuscript in the repository of the Library of the Hungarian Academy of Sciences (http://real.mtak.hu/).
