# Supplementary material for: Vertical optical complexity shaped by submerged macrophytes
Source: Sci Rep. 2024 Mar 1;14:5100. doi: 10.1038/s41598-024-55824-w (PMC10907357; doi:10.1038/s41598-024-55824-w)
Supplement: Supplementary file 1 — Supplementary Figures. [file 41598_2024_55824_MOESM1_ESM.docx]

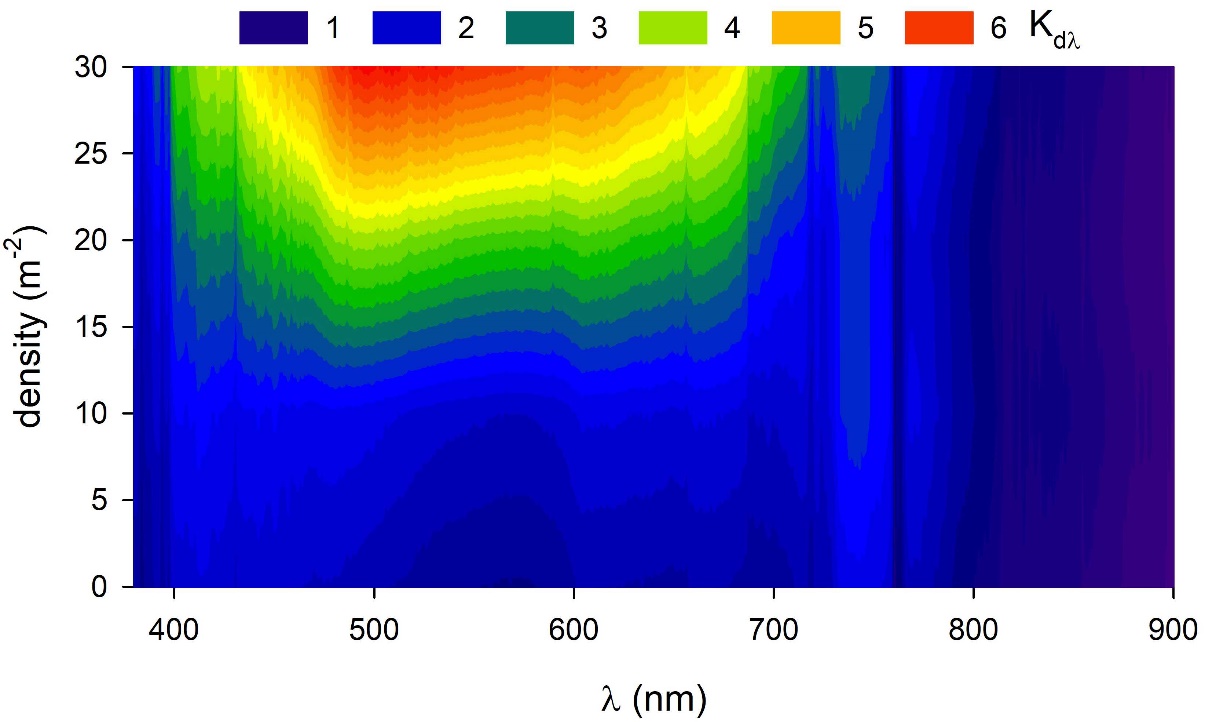


| Supplementary figure 1. Density dependent change of the spectral diffuse attenuation coefficient (K_d(λ)_) in monospecific *Potamogeton perfoliatus* stands in Lake Balaton. | |
| --- | --- |
| 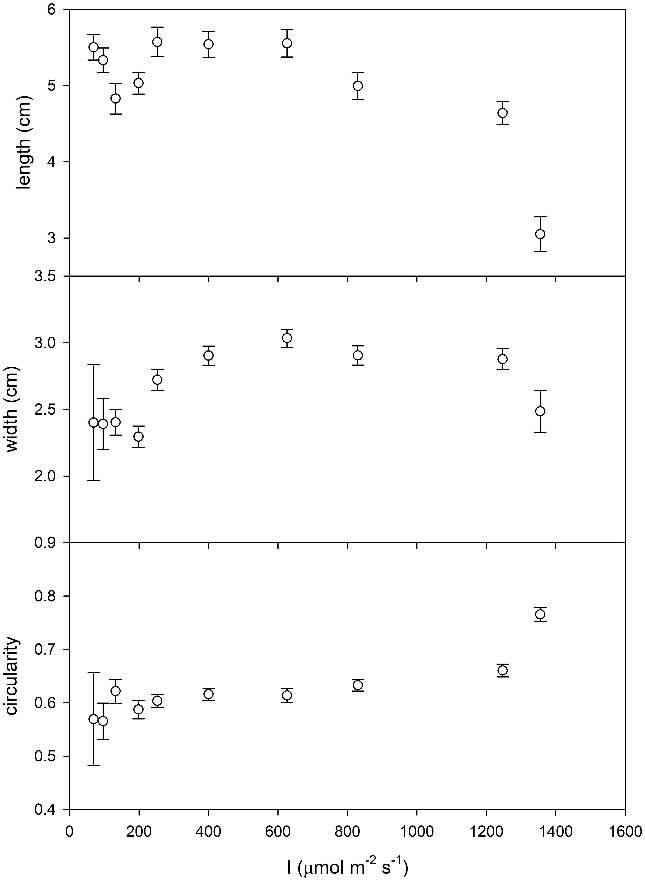 | 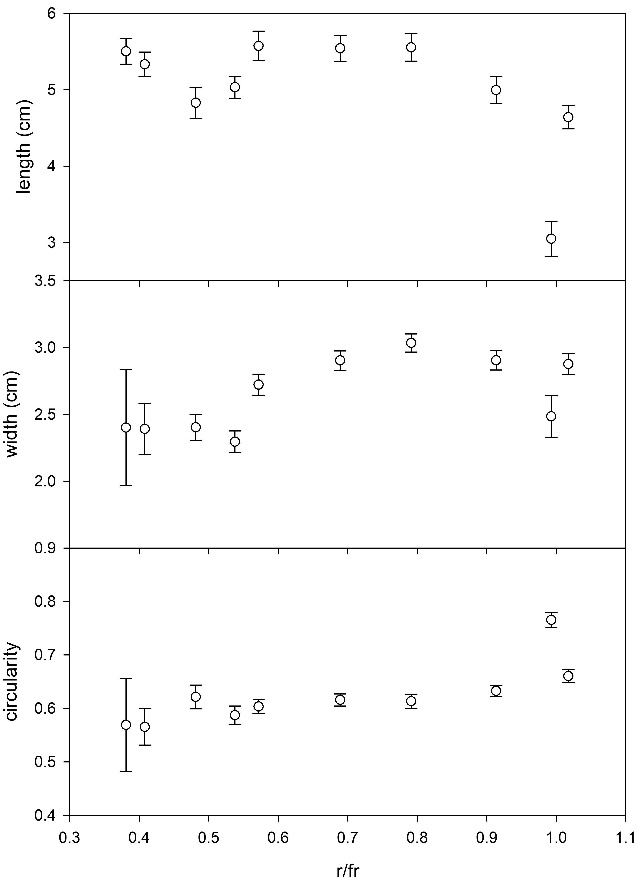 |
| Supplementary figure 2. Effects of light intensity (µmol m^-2^ s^-1^) and red to far red light ratio (r/fr) on morphological parameters of *Potamogeton perfoliatus* in Lake Balaton. | |

| 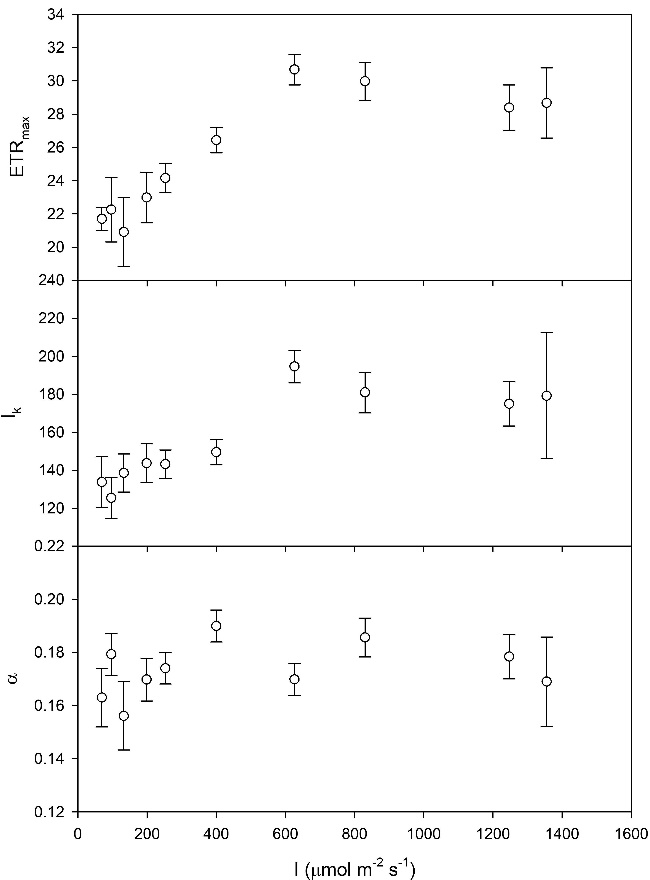 | 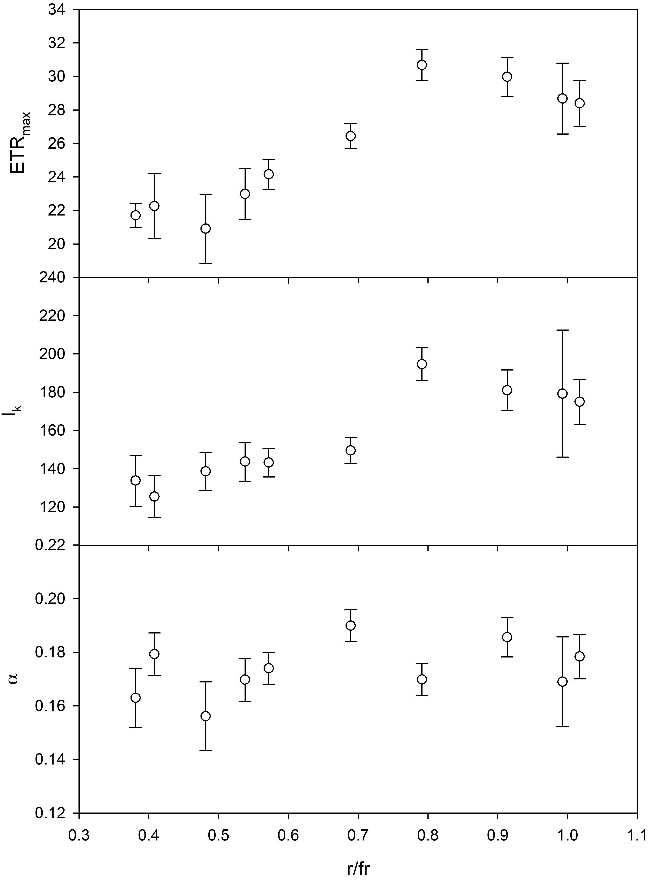 |
| --- | --- |
| Supplementary figure 3. Effects of light intensity (µmol m^-2^ s^-1^) and red to far red light ratio (r/fr) on photophysiological parameters of *Potamogeton perfoliatus* in Lake Balaton. | |

| 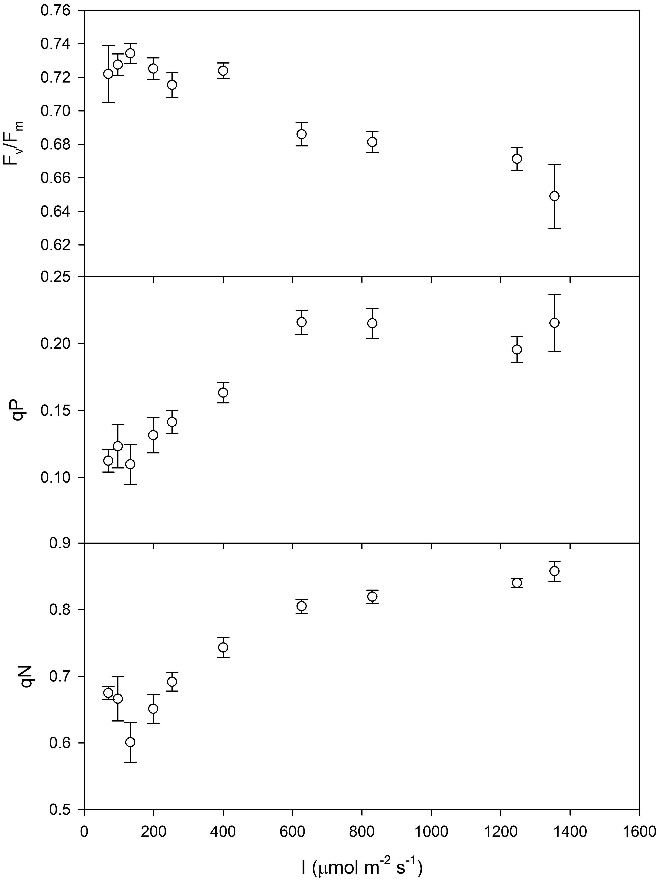 | 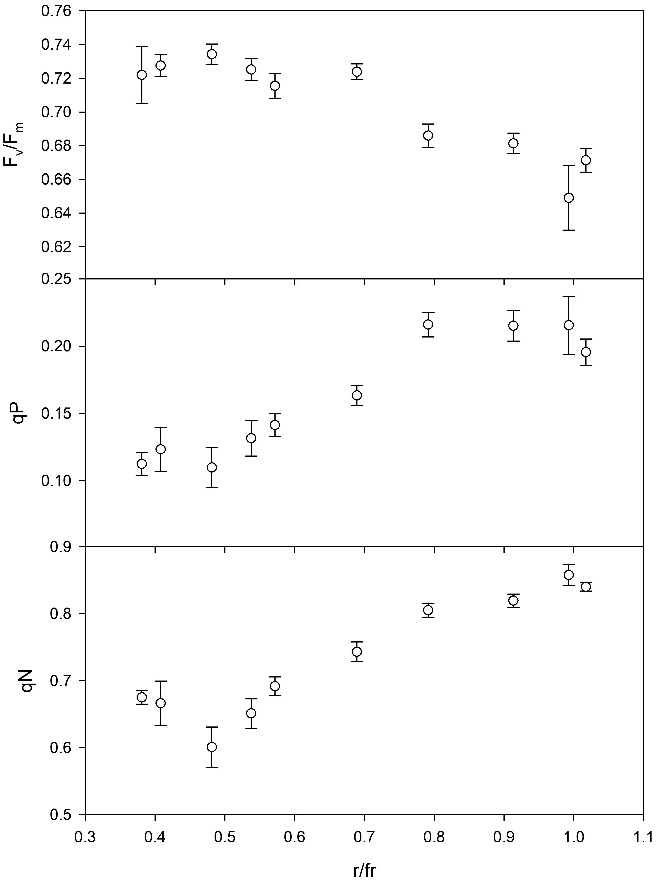 |
| --- | --- |
| Supplementary figure 4. Effects of light intensity (µmol m^-2^ s^-1^) and red to far red light ratio (r/fr) on photophysiological parameters of *Potamogeton perfoliatus* in Lake Balaton. | |

| 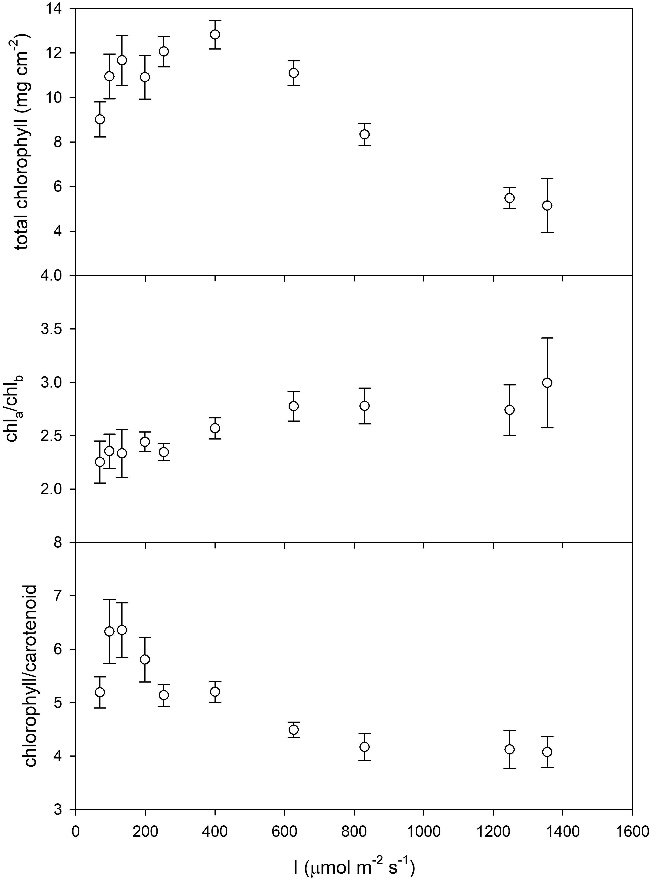 | 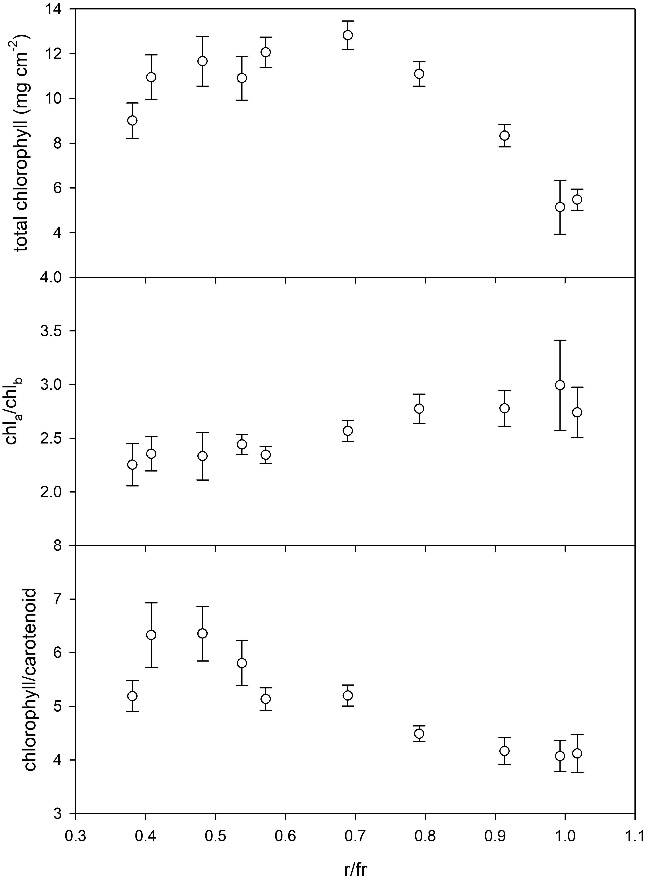 |
| --- | --- |
| Supplementary figure 5. Effects of light intensity (µmol m^-2^ s^-1^) and red to far red light ratio (r/fr) on leaf pigment content of *Potamogeton perfoliatus* in Lake Balaton. | |
